# Co-localized optode-electrode design for multimodal functional near infrared spectroscopy and electroencephalography

**DOI:** 10.1117/1.NPh.12.2.025006

**Published:** 2025-04-08

**Authors:** De’Ja Rogers, Walker Joseph O’Brien, Yuanyuan Gao, Bernhard Zimmermann, Shrey Grover, Yiwen Zhang, Anna Kawai Gaona, Sudan Duwadi, Jessica E. Anderson, Laura Carlton, Parisa Rahimi, Parya Y. Farzam, Alexander von Lühmann, Robert M. G. Reinhart, David A. Boas, Meryem A. Yücel

**Affiliations:** a Boston University, Neurophotonics Center, Department of Biomedical Engineering, Boston, Massachusetts, United States; b Boston University, Department of Electrical and Computer Engineering, Boston, Massachusetts, Unites States; c Boston University, Department of Psychological and Brain Sciences, Boston, Massachusetts, United States; d Boston University, Department of Physical Therapy, Boston, Massachusetts, United States; e Boston University, Department of Speech, Language, and Hearing, Boston, Massachusetts, United States; f Boston University, Questrom School of Business, Boston, Massachusetts, United States; g Technical University of Berlin, Intelligent Biomedical Sensing (IBS) Lab, Machine Learning Department, Berlin, Germany; h BIFOLD – Berlin Institute for the Foundations of Learning and Data, Berlin, Germany

**Keywords:** near-infrared spectroscopy, electroencephalography, methods, high-density diffuse optical tomography, multimodal imaging

## Abstract

**Significance:**

Neuroscience of the everyday world requires continuous mobile brain imaging in real time and in ecologically valid environments, which aids in directly translating research for human benefit. Combined functional near-infrared spectroscopy (fNIRS) and electroencephalography (EEG) studies have increased in demand, as the combined systems can provide great insights into cortical hemodynamics, neuronal activity, and neurovascular coupling. However, fNIRS-EEG studies remain limited in modularity and portability due to restrictions in combined cap designs, especially for high-density (HD) fNIRS measurements.

**Aim:**

We have built and tested custom fNIRS sources that attach to electrodes without decreasing the overall modularity and portability of the probe.

**Approach:**

To demonstrate the design’s utility, we screened for any potential interference and performed a HD-fNIRS-EEG measurement with co-located opto-electrode positions during a modified Stroop task.

**Results:**

No observable interference was present from the fNIRS source optodes in the EEG spectral analysis. The performance, fNIRS, and EEG results of the Stroop task supported the trends from previous research. We observed increased activation with both fNIRS and EEG within the regions of interest.

**Conclusion:**

Overall, these results suggest that the co-localization method is a promising approach to multimodal imaging.

## 1 Introduction

Functional near-infrared spectroscopy (fNIRS) is a noninvasive optical imaging tool that uses near-infrared light to measure the hemodynamic changes associated with the neuronal activity in the brain, with some of its most notable benefits being its spatial resolution^1^^,^^2^ and resistance to electrical artifacts.^2^ With recent developments in fNIRS, such as miniaturized components becoming increasingly available, there has been a proliferation of wearable fNIRS systems both in laboratory research and real-world settings,^3^^–^^5^ and more recently in clinical applications.^3^^,^^6^ High-density diffuse optical tomography (HD-DOT) is an fNIRS technique that has recently increased in popularity due to its improvements in spatial resolution and localization accuracy^7^^–^^9^ compared with sparse fNIRS measurements. Notable limitations of fNIRS are its hemodynamic delay reducing the temporal resolution of the underlying neuronal activity and its depth limitation to the cortical levels of the brain.^1^^,^^2^^,^^10^ Electroencephalography (EEG) is a well-established, noninvasive method of measuring electrical activity in the brain in real time for both research and clinical purposes (i.e., for monitoring patient health and diagnostics),^6^^,^^11^ with the most notable benefit of this method being high temporal resolution and the most notable limitations being low spatial resolution, high sensitivity to noise, and limitation to the cortical levels of the brain.^1^^,^^2^^,^^10^ The benefits and limitations of these two techniques are complementary in nature (i.e., fNIRS having high spatial resolution relative to the low spatial resolution of EEG, EEG having high temporal resolution relative to the low temporal resolution of fNIRS, and fNIRS being resistant to electrical artifacts compared with the extreme sensitivity to electrical artifacts for EEG). So, the use of fNIRS with simultaneous EEG has been explored extensively in the brain-computer interface (BCI) community as a method of increasing the amount of information that can be collected during a single measurement. Through the use of adaptive data analysis methods, this hybridization has also been found to improve the abilities of the individual systems, such as improved temporal response for fNIRS,^12^^–^^14^ improved localization for EEG,^15^ and improved accuracy for both systems.^2^^,^^12^^,^^15^^–^^18^

With both the increased emphasis on high-density fNIRS (HD-fNIRS) arrays and increased interest in multimodal (fNIRS-EEG) imaging, a process that allows for the preservation of electrode and optode arrangements is desirable to ensure sufficient measurement quality in both modalities. This is an important consideration as, with both modalities, the spacing between modules affects the quality of collected signals. Examples of fNIRS-EEG set-ups in past publications often utilize sparse and co-registered arrays, meaning that the fNIRS optodes and EEG electrodes individually require a unique position on the scalp.^2^^,^^19^^–^^21^ As a result, the co-registration of these systems generally requires accepting tradeoffs between coverage, array density, and portability. Therefore, efforts in combining fNIRS and EEG systems via co-localization enable optodes and electrodes to reside on the same cap with significantly reduced space between them, reducing the effects of tradeoffs associated with the combination of these modalities.

Until recently, this combination of systems featured the use of fiber-based fNIRS systems, such as the OxyMon device developed by Artinis Medical Systems, the NIRScout System developed by NIRx Medical Technologies, and the LabNIRS system,^22^^–^^26^ which addressed the compromises regarding coverage limitations. Fiber-based systems unfortunately suffer drawbacks as a result of the weight of the fibers and the bulky laser drivers and detectors used in these systems. This significantly affects system portability and subject range of motion, limiting the use of such fiber-based solutions to constrained lab settings and experimental paradigms. In addition to this, for subjects that are unable to support the weight of a fiber-based system, such as newborns and those with conditions impacting the strength of the neck, a different solution is required. As a result, recent developments in the use of this combined imaging strategy have explored the feasibility of fiberless and wearable fNIRS-EEG system-based studies.^12^^,^^27^^–^^33^ Examples of newer approaches making use of wearable light-emitting diode (LED)-based fNIRS-EEG systems include the Artinis Brite Family^25^ and the Kernel Flow 2 system.^34^

Our goal is to develop fNIRS optodes capable of being co-localized with EEG electrodes and to demonstrate the feasibility of the combined fNIRS-EEG measurements with this new design that addresses the previous limitations of coverage, array density, and portability/wearability. Our design allows for the preservation of both the standardized 05 to 20 EEG arrangement and custom fNIRS probe design, allowing simultaneous HD-DOT and EEG (multimodal HD-fNIRS-EEG) imaging and alleviating the burden of probe modification due to scalp availability and modularity while maintaining the overall portability. The feasibility of this method was investigated through a crosstalk analysis, in which interference of the fNIRS signal on the EEG signal was quantified and evaluated. To further validate this method, 14 subjects completed a modified Stroop task^35^ and the resulting activation detected by fNIRS and EEG were compared with previously published results.

## 2 Methods

### 2.1 Optode Design

**Fig. 1:**
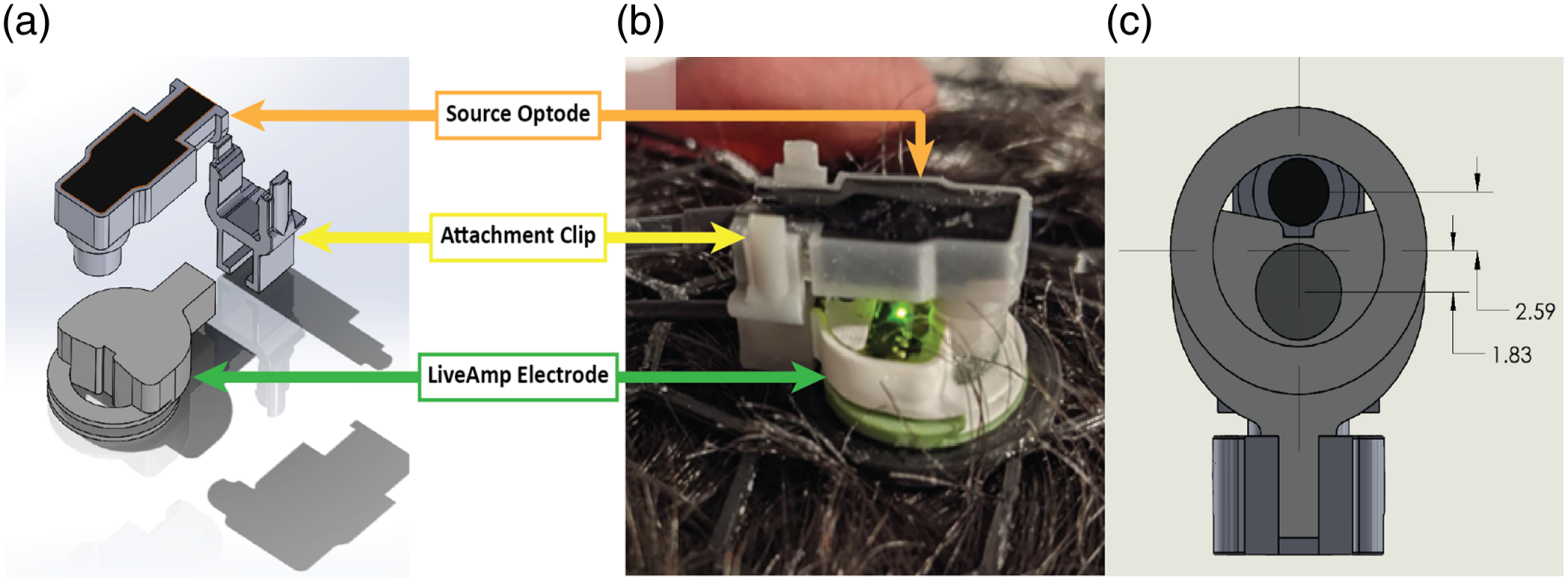
Co-location method. Co-located optode and electrode: (a) separated (design), (b) connected (in practice), and (c) connected, underneath view (design). Dimensions are shown in mm.

To allow for the co-localization of fNIRS optodes and EEG electrodes, a custom optode was designed to mate with the external features of a LiveAmp active wet electrode (BrainProducts, Germany). As shown in Fig. 1, this method enables the optode and electrode to share the same position but maintains separation of the electronics to reduce the possibility of crosstalk in the recorded data. The chosen active wet electrode contains an access hole to enable the application of the conductive gel. This access point was large enough for our 3-mm-diameter light pipes to fit through and contains features specifically designed to ensure that the position of the light pipe remained stable. By mounting the optode directly on top of the active wet electrode and leveraging the access hole on the electrode, the light pipe touches the scalp at a 4.87-mm center-to-center distance from the geometric center of the electrode active contact area (2.59 mm distance from the true center of the electrode housing) as shown in Fig. 1(c).

The EEG-mounted source optodes were fabricated using a selective laser sintering (SLS) 3D-printed resin shell (Form 3, black resin, Formlabs, Somerville, MA, United States). This technique enabled the production of extremely fine-scale features with high resolution and allowed for the use of dielectric epoxy potting to fully isolate the electronics from the electrode gel. The high resolution of the 3D prints allowed for the curvature found at the access hole in the electrode to match, stabilizing the light guide in the desired position. A separate 3D-printed breakaway attachment clip allows for easy insertion and removal of the optode to grant access to the electrode, important for ensuring the experimenter can easily adjust the electrode for better contact with the scalp. The design of the attachment clip was made with the goal of ensuring a secure hold on the optode while simultaneously having a designated weak point to control the location of mechanical failure should it occur so that neither the optode nor the electrode will be damaged from any incidentally applied force (i.e., if the wires are pulled). The gray pro resin (Formlabs, Somerville, MA) used specifically in the breakaway clip provides resistance to failure by the cyclic stresses of insertion and removal of the optode. To ensure good coupling of the light guide to the subject’s scalp, a length of 9.5 mm was chosen to place the tip of the guide ∼1.5  mm below the plane of the electrode bottom.

### 2.2 Experimental Paradigm

#### 2.2.1 Probe Design

**Fig. 2:**
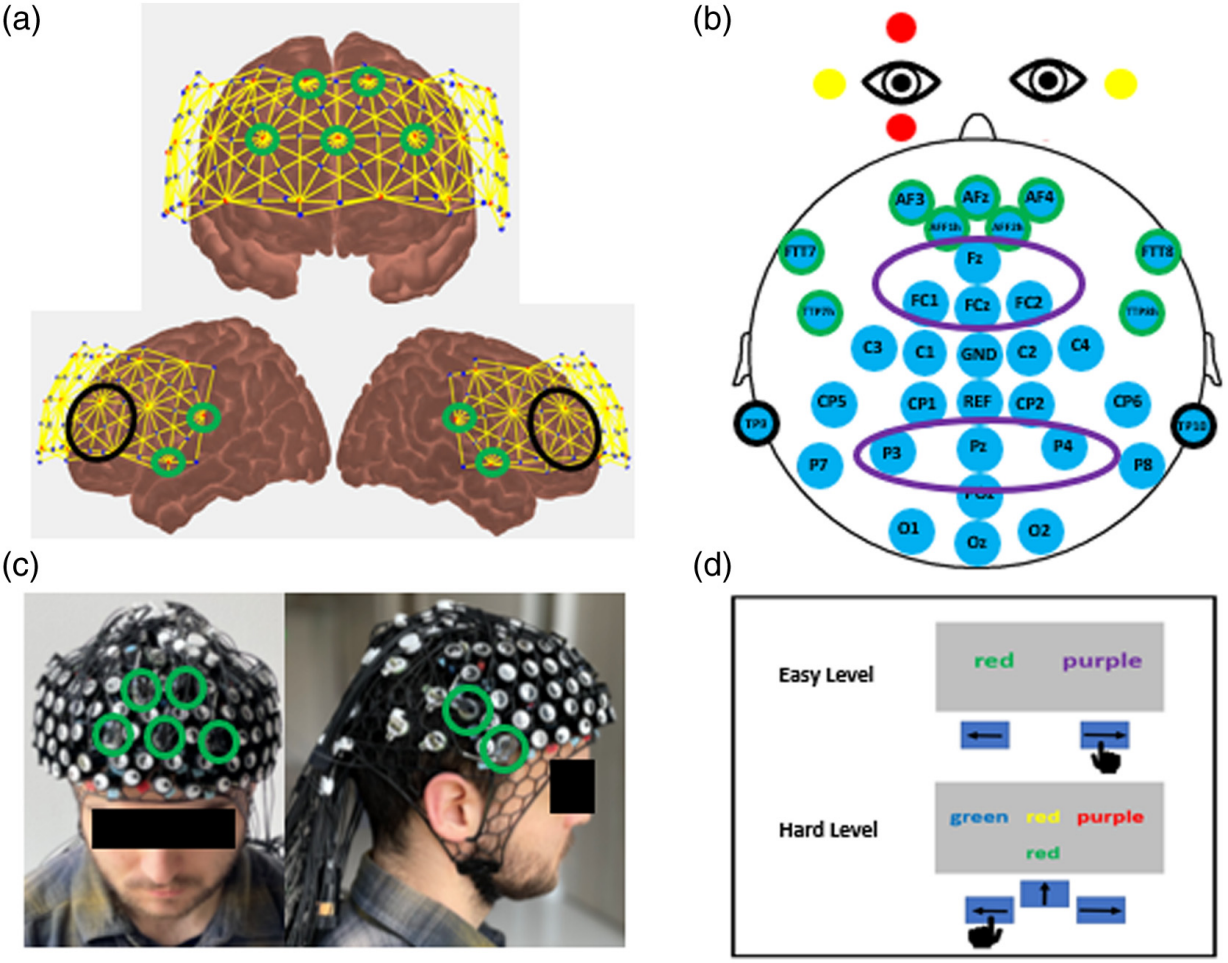
Probe and experimental design. (a) Probe design for fNIRS, where the fNIRS sources and detectors are represented by red and blue dots, respectively. The co-located positions are outlined in green and the fNIRS regions of interest are circled in black (dlPFC). (b) Probe design for EEG, where the EEG positions are represented by light blue circles and the co-located positions are outlined in green. The EEG regions of interest are circled in purple (fronto-central and parietal) and mastoid positions are outlined in black. The horizontal and vertical EOG positions are denoted around the eyes in yellow and red, respectively. (c) Co-location probe in practice, containing the PFC fNIRS and whole head EEG probes combined. The nine co-located positions are outlined in green. (d) Experimental design example for the modified Stroop task.

The HD-fNIRS-EEG probe was designed in the AtlasViewer toolbox.^36^ Optodes were populated across the prefrontal cortex and temporal region, indicated by red for sources and blue for detectors [Fig. 2(a)]. As a grommet type, “NIRX2” was selected to be compatible with optodes from the NIRSport2 systems (NIRx Medical Technologies, Germany). The source–detector optode distances are in the form of a high-density layout, where the first nearest neighbor distances are 8 mm, the second nearest neighbor distances are 19 mm, and the third nearest neighbor distances are 32.5 mm. The electrode positions were populated using various 10-05, 10-10, and 10-20 positions. The grommet type “EBPAS” was selected for the electrodes from the BrainVision LiveAmp system (BrainProducts, Germany). The accelerometer provided by NIRx Medical Technologies was populated right of the Cz position, and the grommet type “NIRX2” was selected for compatibility. The probe design was then converted to a. stl file and 3D printed using NinjaFlex^37^ (NinjaTek, Lititz, PA) TPU material for FDM 3D printing, in our NinjaCap style.^38^ There are 25 sources (nine co-located with EEG), 58 detectors, an additional eight detectors for short separation (8 mm) channels, 32 EEG electrodes (nine co-located with fNIRS), one ground electrode, one reference electrode, and six electrodes dedicated to collecting electrooculography (EOG) data (two ground, two horizontal, and two vertical). The co-located positions are as follows: AF3, AF4, AFz, AFF1h, AFF2h, FFT7, FFT8, TTP7h, and TTP8h (as shown in Fig. 2, panels a, b, and c).

#### 2.2.2 Experimental Design

The study protocol was approved by the Institutional Review Board of Boston University. All participants provided written informed consent to take part in this study. All 19 participants were healthy young adults (mean age: 24±3.5 years, 13 female, one left-handed). We performed a 3-min resting run on five subjects to test crosstalk between fNIRS and EEG systems. During the resting state crosstalk measurement task, subjects were asked to sit and stare at black and white alternating circles in the form of a target on the wall while maintaining an awake and restful position. As the subject sat, the experimenters first ensured that all electrode impedances were less than 10 kΩ. Then, experimenters turned the sources on and off in 10-s intervals and recorded the beginning of the source condition (sources on versus sources off) via manual stimulus marks. The sources were modulated at a frequency of 17.4 Hz, based on a prior understanding of EEG waveforms.^39^ In real time, the EEG signal was observed for any signs of signal distortion. Post-task, a spectral analysis of the task was evaluated to determine whether a 17.4-Hz signal was observed.

To further demonstrate the utility of this design, 14 additional subjects performed a modified version of the Stroop task, adapted from Jahani et al.,^35^ via the 2021.1.4 version of the PsychoPy Presentation Software.^40^ For this task, there were two levels: easy and hard. For the easy level, there were two words on the left and right halves of the screen [see Fig. 2(d)]. Subjects were asked to use the left or right arrow keys on the laptop keyboard to indicate which of the two-colored words on the screen were congruent (the color of the text matches the word meaning). For the hard level, there were four words on the screen, one at the bottom center and three at the top in the left, center, and right positions. Subjects were asked to use the left, right, and up arrow keys to identify which word at the top described the color of the text of the bottom word, regardless of the color in which the top word was written, and irrespective of the actual text of the bottom word. During the rest period on all levels, there was a white cross in the middle of the screen and the rest time was randomized between 10 and 15 s. Subjects completed one run that was ∼10  min, consisting of 18 randomized blocks with six 3-s-long trials per block, and each block consisting of only easy or only hard trials (108 total trials, 54 per condition).

### 2.3 Data Acquisition

To place the cap appropriately for each of the subjects, the protocol is as follows:

The circumference, inion to nasion, and left pre-auricular (LPA) point to right pre-auricular (RPA) point head measurements were recorded. The circumference measurement was used to determine which sized cap to use (55 or 57 cm). The inion to nasion and LPA to RPA measurements were used to find the center (Cz) position on the head, which was then aligned to the center of the 3D-printed cap, which is marked with a 3D-printed cross. In addition to this, we measured and confirmed the distance from the edge of the cap to the nasion, which is reported within our lab to be 1.6 cm, with a less than 0.2 cm margin of error.

#### 2.3.1 Data Acquisition (fNIRS)

Four daisy-chained dual wavelength (730 and 850 nm) NIRSport2 systems (NIRx, Germany) were used for data acquisition. The source multiplexing option in NIRx Aurora Acquisition Software was edited so that multiple spatially distant sources could be on simultaneously to attain an fNIRS sampling frequency of 17.4 Hz. The lab streaming layer (LSL) trigger option in Aurora was used to collect stimulus information from the PsychoPy-based Stroop task presentation. Signal optimization via Aurora software was performed after EEG optimization. The spring tops (strength levels 1, 2, and 3, NIRx, Germany) were used to improve optode-scalp coupling. The hair around the co-located positions was moved using one-sided cotton swabs to maximize optode-scalp coupling while maintaining the quality of the electrode-scalp coupling underneath. The raw intensity data were actively observed during the data acquisition stage to ensure the presence of the LSL trigger and acceptable signal quality. Runs in which subjects had large movements, subjects actively asked questions about the tasks during data collection, or during which technical difficulties occurred (i.e., program crashing or a disconnection of the electronic stimulus) were discarded, and the runs were repeated after a review of the instructions or troubleshooting the technical difficulties.

#### 2.3.2 Data Acquisition (EEG)

The EEG data were acquired at a 500-Hz sampling rate, with real-time filtering option disabled, using a wearable, 24-bit amplifier with 32 standard actiCAP electrodes (BrainVision LiveAmp, BrainProducts, Germany). There were six midline positions (AFz, Fz, FCz, Pz, POz, Oz) and 12 lateral pairs (AF3/4, AFF1/2h, FTT7/8, FC1/2, TTP7/8, C1/2, C3/4, CP1/2, CP5/6, P3/4, P7/8, O1/2) arrayed based on the International 10-05 System. Excluding co-located positions, coordinates were chosen to not overlap with optodes present in the fNIRS design, and embedded in a 3D-printed cap made from NinjaFlex^37^ (NinjaTek, Lititz, PA). The ground electrode was located at Cz, and the reference electrode located at CPz served as the online reference. Signals were re-referenced offline to the average of the left (TP9) and right (TP10) mastoid positions. Combined with this 32-electrode system was a trigger extension and an EOG auxiliary adapter (BIP2 AUX Adapter, gain 100, Brain Products, Germany) to record the stimulus-locked triggers for the given tasks (channel 33) and the EOG signals (channels 34 and 35). The trigger auxiliary channel was connected via a BNC cable to a Neurospec Trigger box, which was then connected to the laptop that sends the PsychoPy presented electronic triggers. The EOG data were recorded using bipolar skin electrodes placed 1 cm lateral to the external canthi to measure horizontal eye movements and bipolar skin electrodes above and beneath the left eye to measure vertical eye movements and blinks [Fig. 2(b)].

### 2.4 Data Analysis

#### 2.4.1 Data quality analysis

To assess data quality, we analyzed the signal-to-noise ratio (SNR) for fNIRS data and impedance levels for EEG data. To evaluate the overall quality of the collected data, we calculated the mean SNR for each subject, confirming that it was higher than the absolute minimum threshold of five. All channels with an SNR of less than 5 were excluded from the analysis. The mean SNR across all subjects was calculated. In addition, we calculated the percentage of non-co-located, co-located, and total channels that were pruned compared with the total number of channels. When considering the EEG data, all electrode positions were optimized to maintain impedance levels below 10 kΩ throughout the study. To evaluate EEG signal quality, we calculated the mean impedance for each subject and confirmed it was below the absolute maximum threshold of 10 kΩ. The mean impedance across all subjects was calculated.

#### 2.4.2 fNIRS data analysis

The raw fNIRS data was analyzed using the 1.89.0 development version of the Homer3 software.^41^ All channels were preprocessed using the hmrR_PreprocessIntensity_Negative function to remove potential negative values. Channels were classified as noisy if their intensity values were below 0.002 arbitrary units (AU) or if their signal-to-noise ratio (SNR) was below the threshold of five. Those noisy channels were then pruned using the hmrR_Prune Channels function. Next, the data were converted from intensity into the change in optical density using the hmrR_Intensity2OD function. Using the hmrR_MotionCorrectSplineSG function, motion artifacts were corrected by combining the Spline Interpolation and Savitzky–Golay Filtering methods with the standard degree of the spline function being 0.99 and a 10-s step length.^7^^,^^42^ Next, any residual slow motion artifacts, which were exaggerated by the SplineSG function, were identified using the Homer3 hmrR_MotionArtifact function, with a standard deviation threshold of 60, an amplitude threshold of 0.4, and masks half a second around the origin of the motion artifact. Afterward, these identified motion artifacts were removed using the hmrR_StimRejection Homer3 function, which removed −2 to 20 s of time around the trial that contained the motion artifact. Then, the data were low-pass filtered at a threshold of 0.5 Hz, using a Butterworth filter with a filter order of 3, and converted from change in optical density to concentration using the hmrR_OD2Conc function with a partial pathlength factor of 1 for both wavelengths. Finally, using the hmrR_GLM function, the hemodynamic response function, for the time range of −2 to 30 s, was modeled using Gaussian functions with a standard deviation of 1 s, their means separated by 1 s, and a short separation regression threshold of 15 mm, where the second (19 mm) and third (32.5 mm) nearest neighbor channels regress out the first nearest neighbor (8 mm) with the greatest correlation. The weights of the regressors were obtained using ordinary least squares fit. The resultant hemodynamic response functions (HRFs) were then exported, and the channels in the region of interest (ROI) were averaged and statistically compared across easy and hard conditions.

The ROIs, defined as regions of expected activation during the Stroop task, were determined to be the dorsolateral prefrontal cortex (dlPFC) by previous research.^43^^–^^46^ The AtlasViewer channels were projected onto the cortex, from which we identified the MNI coordinates. Those channel coordinates were then cross-referenced with the BioImage Suite Website^47^^,^^48^ to confirm the channels in the region of interest, the dlPFC. Channels that were not initially labeled as a dlPFC channel, but were surrounded by dlPFC channels, were included in the ROI. In addition, channels that were labeled as left or right dlPFC on one side of the probe, but not the other, were included in the ROI, as the probe is symmetrical. After cross-referencing the MNI coordinates from AtlasViewer with that of the BioImage Suite Website, twenty channels (10 left and 10 right) remained in the ROI. The channels used in the channel space analysis are as follows: source 15 to detectors 44, 45, 46, 47, and 56; source 23 to detector 56, source 24 to detectors 32, 45, 46, and 56; source 11 to detectors 3, 37, 38, 39, and 52; source 19 to detectors 27, 37, 38, and 52; and source 20 to detector 37. These channel locations are roughly indicated in Fig. 2(a). A paired t-test was performed comparing the HRFs of the conditions (easy versus hard) from 10 to 25 s after the stimulus, using the standard alpha value of 0.05. The effect size for this same averaged data was also calculated. A paired t-test of the individual conditions with rest and their corresponding effect sizes were also calculated.

DOT image reconstruction was performed using the development version (V2.48.1) of the AtlasViewer software.^36^ The results from the Homer3 software were loaded into the AtlasViewer. MCXlab^49^ was used to generate the brain and scalp sensitivity profiles. Then, the image reconstruction was performed on the easy and hard conditions, over the range of 10 to 25 s. The regularization parameter, alpha, was set to 0.01. Afterward, the image space t-statistics for the easy level, hard level, and difference between the two were calculated by converting the hemodynamic concentrations back to optical density for each subject, completing image reconstruction, and conducting a t-test in image space for the conditions separately and a paired t-test to compare the two conditions with each other, using the standard threshold of 0.05. The results were plotted onto the AtlasViewer brain mesh using the image reconstruction methods previously stated.

#### 2.4.3 EEG Data Analysis

##### Crosstalk Analysis

A MATLAB script was developed to analyze the crosstalk runs, consisting of six trials for each manually applied stimulus mark (“Source On” and “Source Off”). For each condition and co-located channel, the 10-s epochs were averaged. Trials were excluded if there were not at least 10 s of data after the manually applied stimulus mark. Following this, a power spectral analysis^50^^–^^52^ was conducted and plotted, initially at the subject level and subsequently at the group level. At the group level, the peak power density for both conditions at ∼17.4  Hz and the maximum peaks within the crosstalk frequencies of interest (16 and 18 Hz) were extracted and compared. A t-test comparing the co-located and non-co-located positions in the “Sources On” condition, for the final iteration of the modified optode, was computed.

##### Stroop Task Analysis

The raw EEG data were analyzed using the MATLAB-based Fieldtrip Toolbox.^53^ Each run of the subject data was loaded, demeaned, detrended, and bandpass filtered between 1 and 45 Hz, to remove any screen noise and potential interference from other devices. Next, the stimulus-locked trials were defined and any trials with incorrect behavioral responses and trials with a standard deviation greater than 20 or a variance greater than 200 were excluded from the results. Then, independent component analysis (ICA), components for which investigation yielded common noise and motion artifacts, was rejected. Channels with poor signal quality were removed and interpolated. ERPs were averaged, plotted, and observed for the P300^54^^–^^58^ component of interest and the EEG regions of interest for the Stroop task [i.e., fronto-central (Fz, FCz, FC1, FC2, C1, C2, C3, and C4) and parietal (Pz, P3, P4, CP1, and CP2)]. The latency of interest was determined similarly to that of conventional latency calculations for EEG.^59^^,^^60^ Significant epochs were defined in a two-part condition: (1) a period during which the difference wave deviated from baseline by greater than 2 standard deviations for longer than 50 ms, (2) provided it exceeded 3 standard deviations in that interval. The beginning of the significant epoch serves as the onset time for the latencies of interest. When the two conditions no longer meet, the 50 ms time point after the last defined significant epoch serves as the offset time for the latencies of interest. A paired t-test was calculated for the onset times, peak amplitude values, and average amplitude across the latency period, for each region of interest. More detailed analysis methods are in Sec. 6.

## 3 Results

### 3.1 Crosstalk Results

**Fig. 3:**
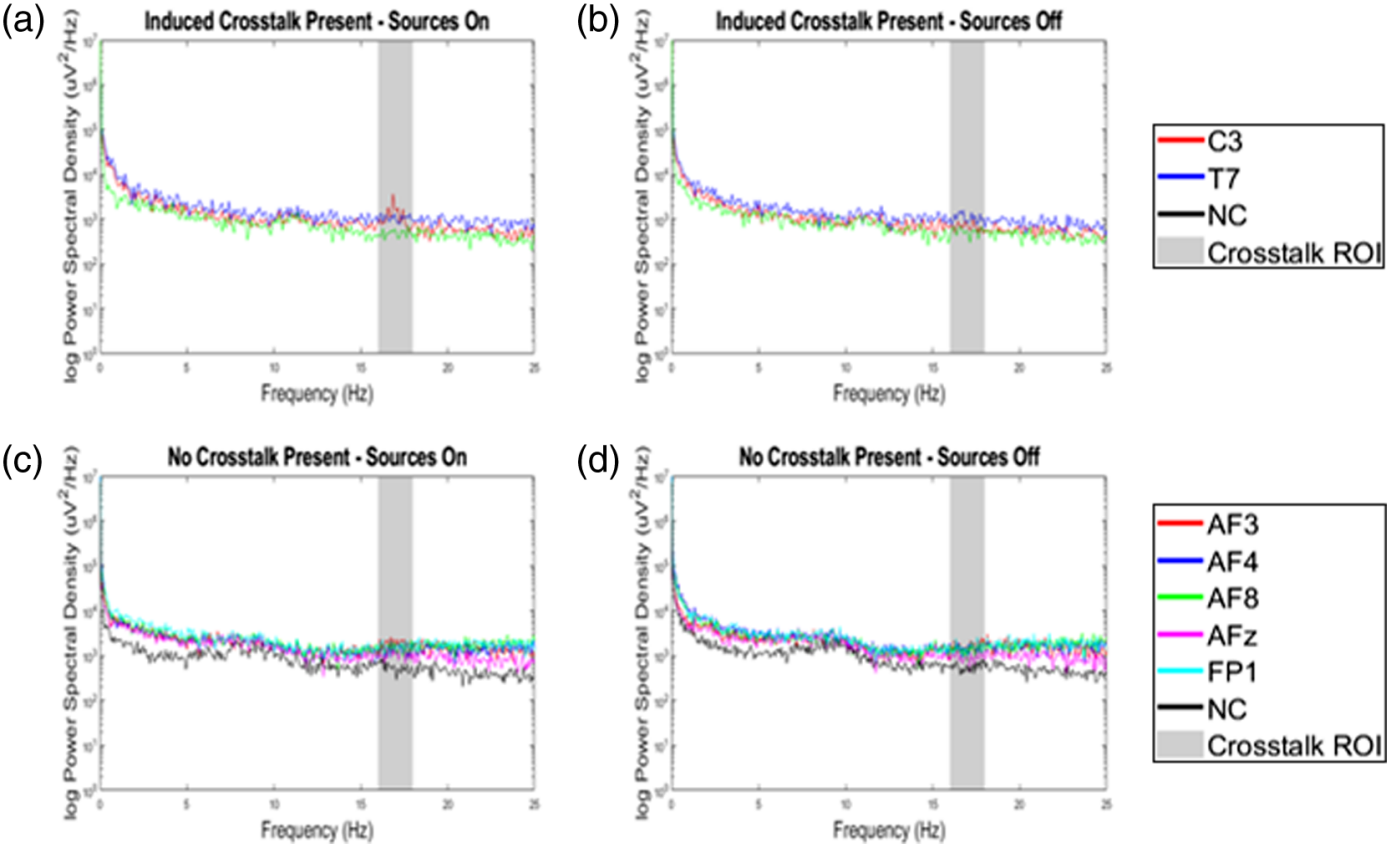
Crosstalk experiment. Example of spectral analysis in which crosstalk was (a) apparent (one subject with a faulty optode) with fNIRS sources on versus (b) off and (c) not apparent (average of 5 subjects) with sources on versus (d) off, at the source modulation frequency (17.4 Hz). This is represented by the shaded area between 16 and 18 Hz. NC represents the average of all non-co-located positions, and all other abbreviations represent 10-05 locations. The peak between 8 and 12 Hz is common, as it represents the alpha wave range that can occur during an eyes closed and/or relaxed state.

Figures 3(a) and 3(b) display the comparison of the spectral analysis of the averaged EEG signals at the co-located and non-co-located positions for one pilot subject, who participated during a previous iteration of the modified optode. These two plots compare the peak spectral power at the source modulation frequency of 17.4 Hz when the sources are on [Fig. 3(a)] and off [Fig. 3(b)]. This is an example of the presence of crosstalk, as the induced interference is shown at the co-located position, C3. At the C3 position on the “Induced Crosstalk Present – Sources On” graph [Fig. 3(a)], the power density at ∼17.4  Hz is $1.37\;{\mathrm{mV}}^{2}/\mathrm{Hz}$, and the same position on the “Induced Crosstalk Present – Sources Off” graph [Fig. 3(b)] shows $0.96\;{\mathrm{mV}}^{2}/\mathrm{Hz}$. By contrast, the “No Crosstalk Present” graphs are of five different co-located positions and the average of all non-co-located positions, averaged from five different subjects recruited later in the study, with the latest iteration of the modified optode. At the group level, the “No Crosstalk Present – Sources On” graph [Fig. 3(c)] and the “No Crosstalk Present - Sources Off” graph [Fig. 3(d)] show no visual peaks resulting from the fNIRS sources’ interference with the EEG signal. The numerical values of the positions for the “Induced Crosstalk Present” graphs by trial and the numerical values for the positions for the “No Crosstalk Present” graphs by subject can be found in Tables 2 and 3 in Sec. 6.3, respectively. The t-test of the co-located and non-co-located positions in the “No Crosstalk Present Sources On” graph resulted in a p-value of 0.51.

### 3.2 Behavioral Results

**Fig. 4:**
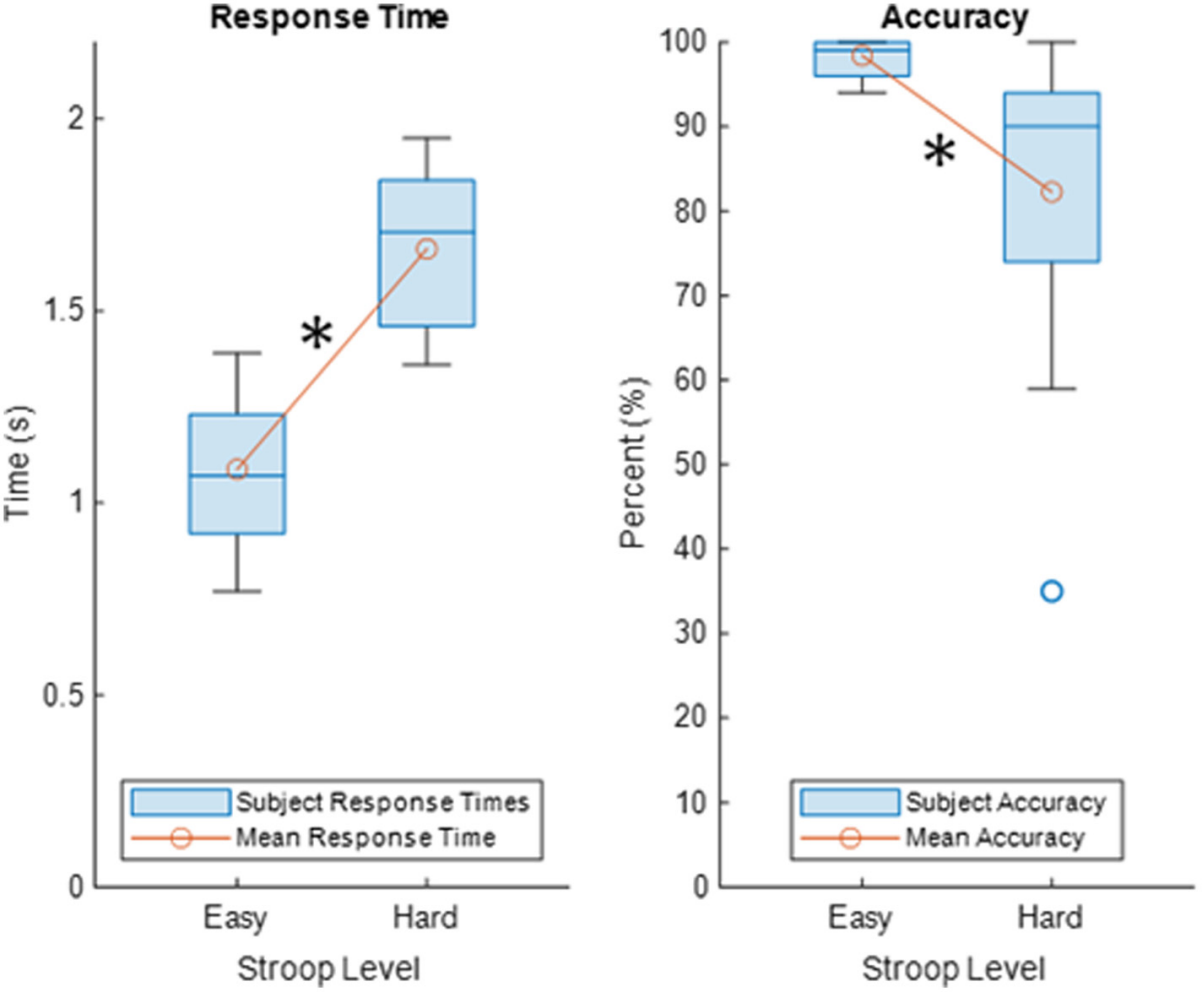
Behavioral results. Boxplots of the response times (left) and accuracy (right) of the 14 subjects, with the average response time and average accuracy represented as red circles connected by a red line. The black asterisk indicates a statistical significance at the 0.05 alpha threshold.

Figure 4 displays a boxplot of the group average response time (left) and accuracy (right) for the fourteen subjects in this Stroop task study. The group behavioral results for the fourteen subjects indicate that the hard level of the modified Stroop task showed increased response time and decreased accuracy compared with the easy level (paired t-test p-values p<0.001 and 0.004 for the response time and accuracy, respectively).

### 3.3 Data Quality Results

Regarding the investigation of fNIRS data quality, 16/25 source optodes are non-co-located and 9/25 source optodes are co-located, so there are ∼16/9 more non-co-located channels than co-located in this probe design. Across all subjects, the mean SNR was ∼42±17 (n=14). When investigating the pruned channels, ∼27±9% (9±3% attributed to co-located and 18±7% attributed to non-co-located channels) of all 238 channels were pruned (Tables 4 and 5 in Sec. 6.3). One participant’s data were removed due to an electronic trigger error; hence, the mean impedance was taken across 13 subjects. The mean impedance was ∼4±1.6  kΩ (n=13). Regarding the investigation of EEG data quality, there are 30 electrode channels in total. For three subjects, noisy channels were marked, removed, and then interpolated using surrounding channels and all channels were co-located positions. The following channels were marked as noisy: AF4 (marked for subject SS025), AFz (marked for subject SS025), AF3 (marked for subjects SS025 and SS028), and FTT8 (marked for subject SS024). Aside from the re-referenced mastoid positions (TP9h and TP10h), all EEG channels were used in group analysis.

### 3.4 fNIRS Stroop Results

**Fig. 5:**
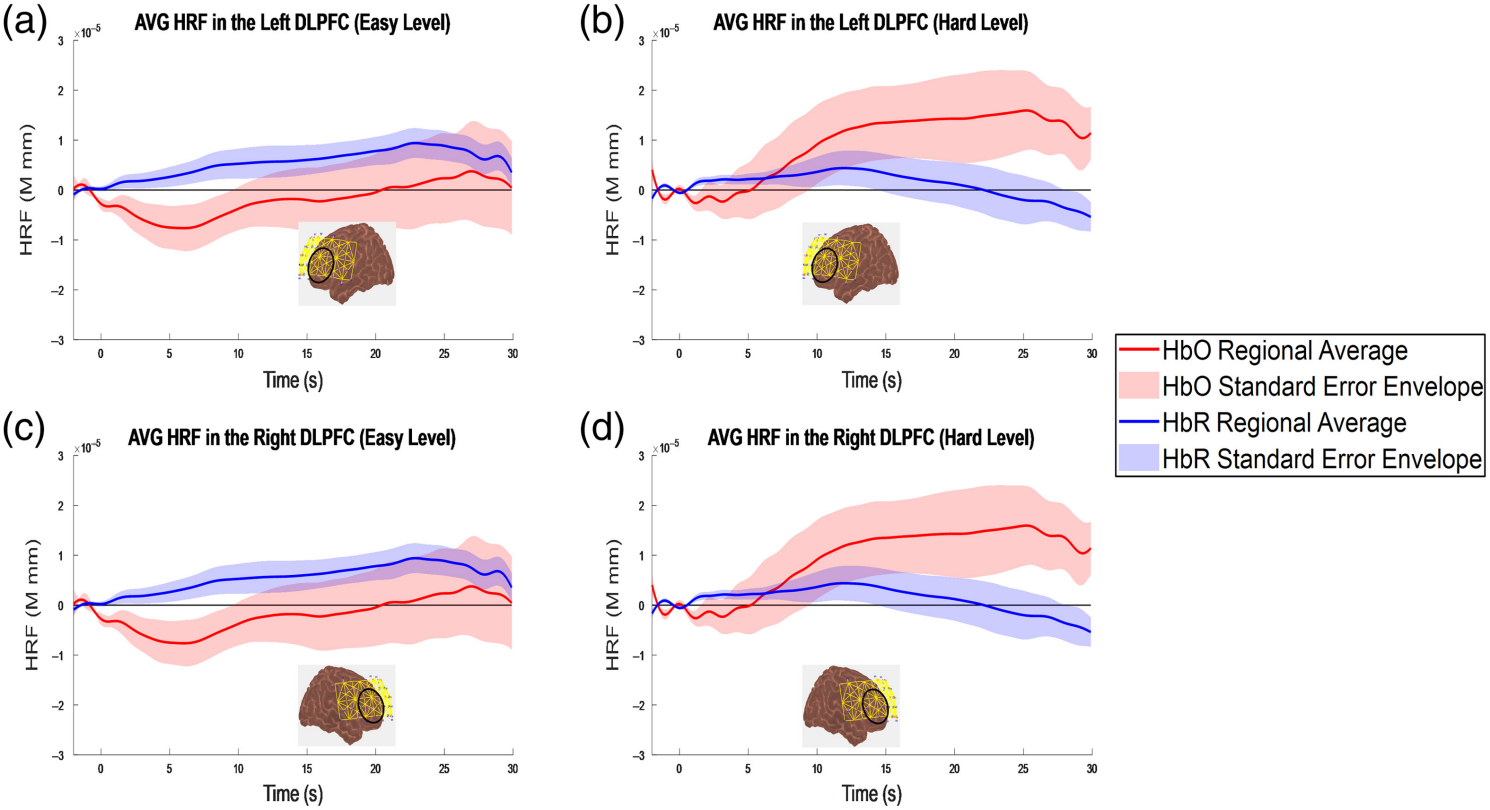
Channel space results: Group Hemodynamic response in the Stroop task regions of interest for 13 subjects. The plots show (a) the left DLPFC at the easy level, (b) the left DLPFC at the hard level, (c) the right DLPFC at the easy level, and (d) the right DLPFC at the hard level. The portion of the probe outlined in black at the bottom of each graph represents the region of interest for that specific graph.

The fNIRS channel space and image space results are shown for 13 subjects. One subject’s data were excluded due to the corresponding hard-level percent accuracy from EEG analysis. Figure 5 shows the group average HRF by region of interest. A paired t-test of the comparison between the two conditions (i.e., easy and hard levels of the Stroop task) shows the p-values of the HbO for the left and right regions of interest being 0.09 and 0.37, respectively. The corresponding effect sizes for the left and right regions of interest are 0.49 and 0.37, respectively. The results of the paired t-test for the HbR for the comparison between the two conditions for the left and right regions of interest are 0.20 and 0.08, respectively. The corresponding effect sizes for the HbR comparison of the two conditions for the left and right regions of interest are −0.25 and −0.52, respectively. None of the changes between conditions are significant. The results of the paired t-test for the individual conditions compared with the rest are in Table 8 in Sec. 6.3 and show that only the HbR at the easy level for both the left and right regions of interest are significant. All statistics were calculated using the average hemodynamic response from 10 to 25 s.

**Fig. 6:**
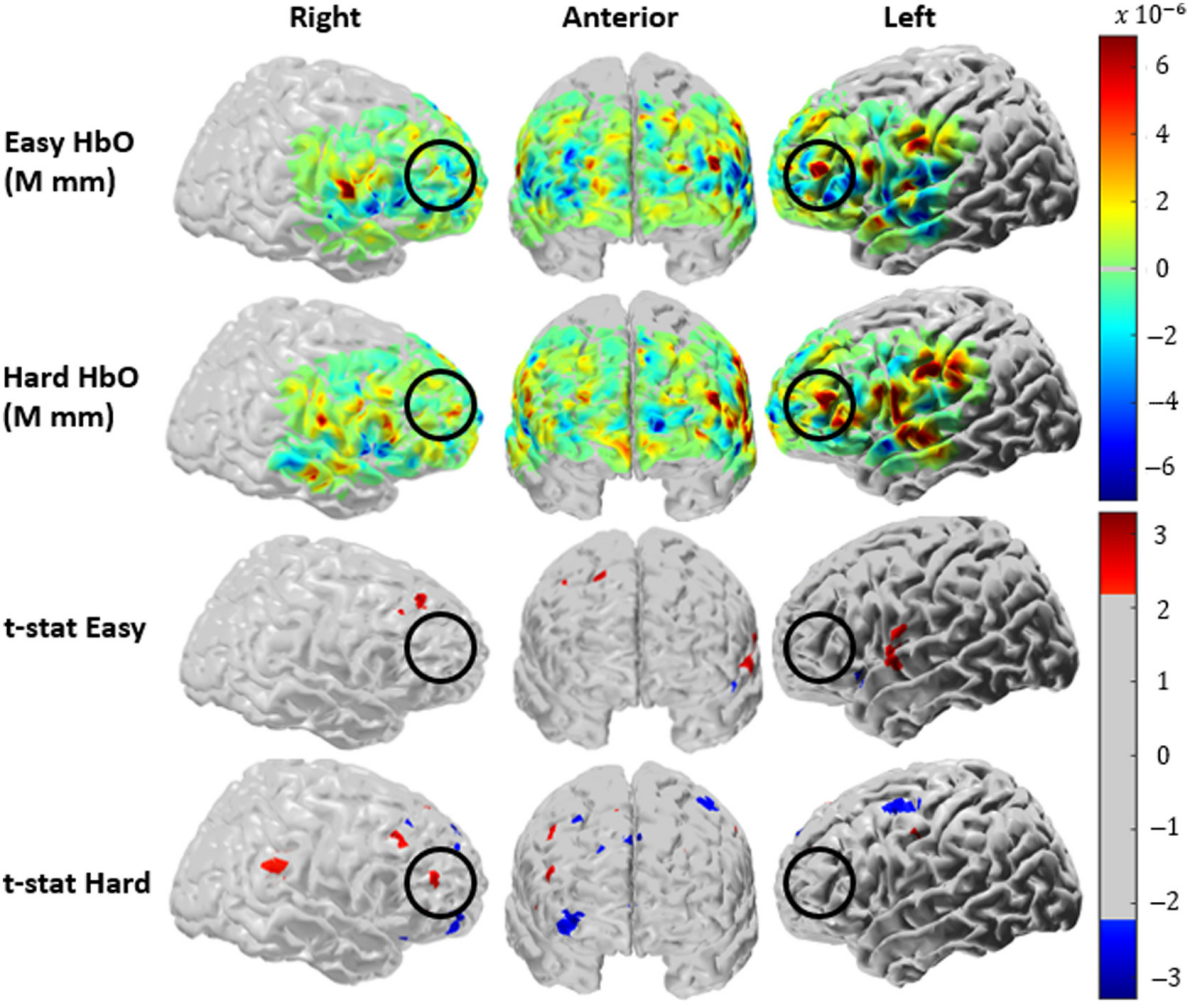
Image reconstruction results: The image reconstruction of the average hemodynamic response across the 10 to 25 s timespan for the easy (first row) and hard (second row) levels of the Stroop task from 13 subjects. The bottom two rows of figures represent the t-statistic of the easy (third row) and hard (fourth row) level conditions overlayed on the brain. The portion of the probe outlined in black represents the regions of interest. The colormap range on the top right is for the easy and hard level conditions and the colormap range on the bottom right is for the t-statistic of the easy and hard level conditions.

The statistical t-maps (Fig. 6, bottom two rows) showed a localization of oxygenated hemoglobin in the dlPFC and temporal regions. There was a visual increase in oxygenated hemoglobin from the hard level, compared with the easy level, for the bilateral dlPFC which is the indicated region of interest outlined in black (Fig. 6 top 2 rows). The t-statistics yield significance in the hard level for the right DLPFC (Fig. 6, fourth row).

### 3.5 EEG Stroop Results

The EEG ERP results are shown for 10 subjects. Four subjects were excluded from the EEG data analysis (1 due to an electronic trigger error, 1 due to the hard level accuracy ratio, and 2 based on the trial exclusion ratio). The rationale for the removal of the data for the two subjects with a poor trial exclusion ratio is that subjects that have many trials removed even after completion of the post-processing steps can be an indication of noisy data. Because the group level averages the averaged subject level data, the subject files that were marked as noisy were excluded, to not average noise into the overall results.

**Fig. 7:**
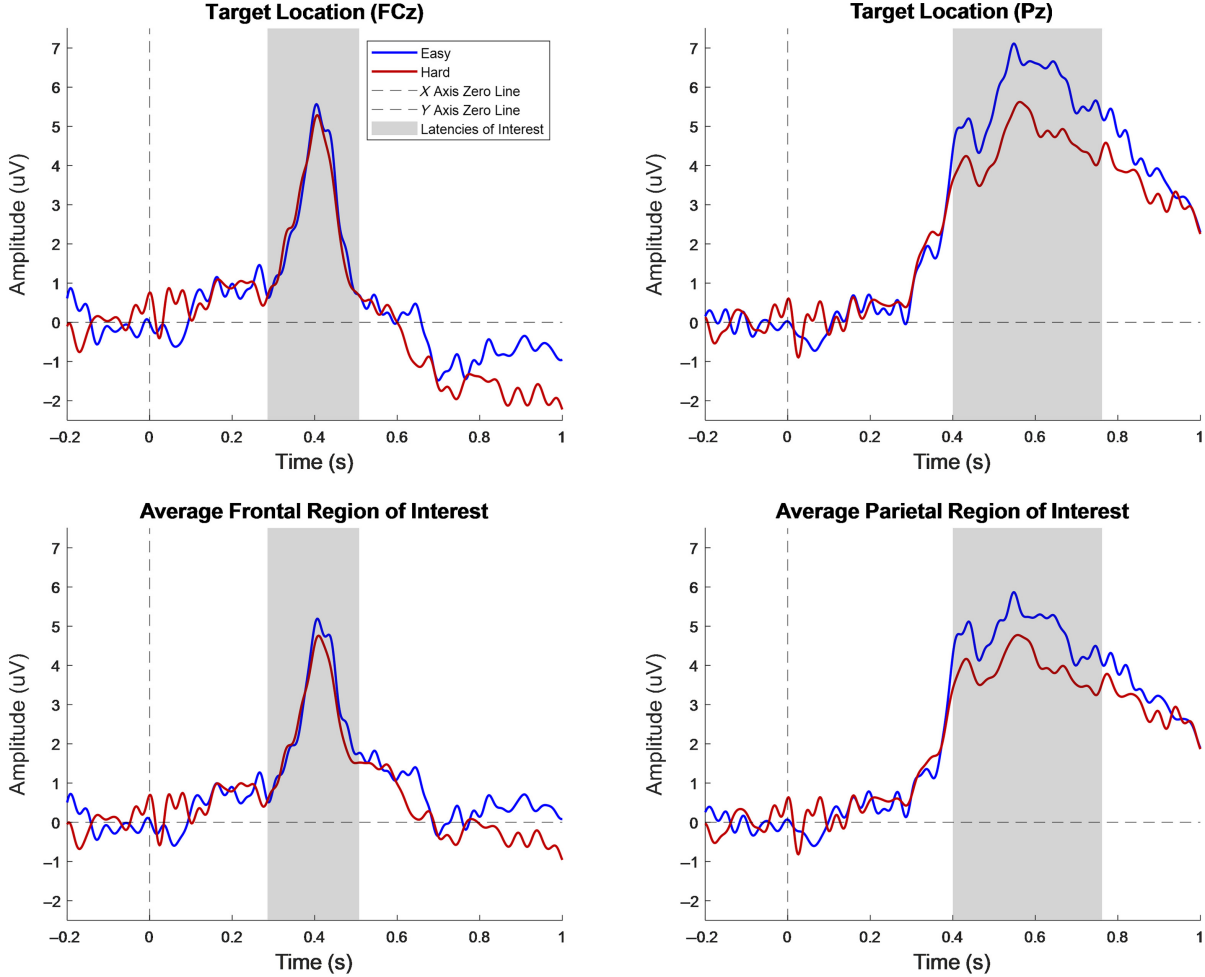
Group average EEG event-related potential results for 10 subjects. The stimulus-locked even–related potential in electrodes in the EEG Stroop task regions of interest, the frontal-central (left) and the parietal (right) regions. The signal colored in blue represents the easy level, and the signal colored in red represents the hard level of the Stroop task. The dashed black lines indicate the X- and Y-axis zero lines. The shaded regions indicate the P3a and P3b latencies of interest.

The calculated onset and offset times for P3b were based on the Pz difference wave and had values of 400 and 762 ms, respectively. Because the difference wave for FCz produced no significant epochs, the calculation methods were applied to the easy and hard levels separately and the earliest onset time and latest offset time were used as the latency of interest for FCz and the fronto-central region. The onset and offset times were 286 and 508 ms, respectively. There was a presence of P3a in the FCz and the fronto-central region, with little visual difference between the two conditions. There was a presence of P3b at the Pz electrode location and in the parietal region, with the easy level being higher in magnitude. Figure 7 shows the ERPs of the target electrodes and averaged ROIs. Table 1 shows the latency, amplitude (peak and average), t-statistic, and effect sizes (which were calculated based on the average) for each region of interest, with all numerical values being truncated after the hundredth place. Table 1 statistical results are of the comparison between the easy and hard Stroop conditions. The t-statistic based on the peak and onset times can be found in Tables 6 and 7 in Sec. 6.3, respectively.

**Table 1:** EEG statistical results: This table shows the peak and mean averages of the easy and hard level conditions by region and latency, as well as the t statistic and effect size for each region and latency, comparing the easy and hard Stroop conditions.

ROI	Latency name	Latency (ms)	Easy level peak amplitude (uV)	Hard level peak amplitude (uV)	Easy level mean amplitude (uV)	Hard level mean amplitude (uV)	t-statistic (mean)	p-value (mean)	Effect size (mean)
FCz	P3a	286 to 508	5.56	5.37	2.79	2.67	−0.14	0.88	0.04
Pz	P3b	400 to 762	7.11	5.38	5.81	4.41	−1.43	0.18	0.45
FC Region	P3a	286 to 508	5.19	4.87	2.79	2.54	−0.33	0.74	0.10
P Region	P3b	400 to 762	5.86	4.58	3.84	3.86	−1.11	0.29	0.35

## 4 Discussion

In this work, we have shown that the high-density co-localization of fNIRS optodes with EEG electrodes is feasible and we have showcased its potential for use. We have addressed the potential limitations of combining HD-fNIRS and EEG in studies by showing how scalp space can be optimized without interference from the source optodes onto the EEG signal. We have also shown that the signal quality of both systems is not adversely affected by the co-location method and that we can reproduce similar findings for a common cognitive load task, such as the Stroop task, to previous research.

### 4.1 Crosstalk

Preliminary results showed a subtle presence of crosstalk in one of two locations for one of three subjects. The modified optode that was responsible for the observed crosstalk had a manufacturing defect due to trapped air in the epoxy forming a large void that reduced the thickness of the dielectric layer to under 1 mm compared with 4 mm of the other optodes. The potting compound in this optode still provided a barrier insulated from direct contact with the conductive gel for the electrode so initially it was believed that performance would not be impacted; however, this void allowed excess gel applied to the electrode to be in close proximity to the area of the PCB directly above the location of the LED. Following the identification of this issue the void was filled and crosstalk was reduced, but for the following subjects, it was decided to replace this optode entirely as a precaution. After improved hardware, the crosstalk task was repeated on five subjects, in five locations. The results of this study, post-improvements, show that there was no observable change in the raw signal between conditions (source on and source off) as compared with that of the faulty optode. The spectral analysis at each location showed no peaks at the 17.4-Hz modulation frequency; therefore, we conclude that there was no significant presence of interference in the collected EEG signal. The numerical analysis of these comparisons supports this finding, as it shows that the spectral analysis of both co-localized and non-co-localized electrode positions indicate that the peaks in the spectral data vary across all locations (both co-localized and non-co-localized) regardless of the source condition (on or off). The only exception is the co-localized position, C3, from the “Induced Crosstalk Present” graphs. For this position, the “Sources On” condition consistently showed the spectral peak at 17.4 Hz, whereas the peak of the “Sources Off” condition for this position varied, like that of all the other positions. The t-test performed on the co-located and non-co-located positions from the “No Crosstalk Present” graphs show that there is no significant difference between the positions, further confirming the lack of interference of the source optodes on the EEG signal at the co-located positions. Overall, the co-location of source optodes and electrodes has been successfully and consistently recorded without the presence of crosstalk.

### 4.2 Signal Quality Results

The data quality analysis for the fNIRS data showed that the average SNR for all subjects above the threshold and co-located channels made up approximately one third of the total channels pruned due to poor signal quality, caused by poor optode-scalp coupling. As the co-located channels made up approximately one third of all channels, this indicated that the co-located channels were no more likely to be pruned than non-co-located channels. The data quality analysis for EEG data showed the mean across and within subjects, as well as all electrodes being below the 10 kΩ impedance threshold. The four noisy channels that were marked (AF3, AF4, AFz, and FTT8) for three of the ten subjects were co-located positions, with three of those four (AF3, AF4, and AFz) being located in the medial PFC and all three of those positions being marked for the same subject. Upon further investigation, user feedback reported high difficulty in maintaining good optode scalp-coupling in the medial PFC, where five of the nine co-located positions were located, due to limited spacing available to maneuver hair with the HD-fNIRS-EEG probe design. Therefore, it was concluded that pruning and marking of noisy electrode channels in that region was most likely due to the limited spacing for hair maneuvering and not the co-location method specifically, meaning that the co-location method did not negatively affect the fNIRS and EEG data quality. To address the challenge of limited hair maneuvering space, we began maneuvering the hair through the NinjaCap before applying electrode gel for EEG or attempting fNIRS optimization. This step took ∼40  min to complete and allowed us to efficiently move and keep hair away from the optode and electrode contact points, which both reduced the overall set-up time and improved signal quality through increased electrode-scalp coupling for other incoming subjects.

### 4.3 Design Considerations

Limitations of the co-location design include the potential reduction of optode-scalp coupling for co-located positions, a long set-up time of ∼2  h, and the potential effect on EEG signal quality. User feedback for the co-location method references other established methods to increase optode scalp coupling, such as spring tops created by NIRx Medical Technologies,^26^ though our method lacks these techniques. As a result, the method used to increase optode-scalp coupling, using a cotton swab to maneuver hair, became ineffective, when pressure was the reason for reduced coupling. Future work to address this limitation includes designing and implementing an improved module design that adds this spring-loaded mechanism, offering the same co-located functionality but improving the scalp coupling of the light guiding element if needed.

The HD-fNIRS-EEG cap consisted of 238 fNIRS channels and 30 electrodes, causing the set-up time for this study to be ∼2  h. The participants were required to sit patiently during this 2-h time block, which often left them in a more relaxed, near drowsy state upon completing the tasks. This could explain the alpha waves that were locked into the EEG results despite averaging, filtering, and other processing methods. Future work would include the optimization of the set-up process through more experimenters and alternate methods to keep the participants awake and alert, such as the inclusion of drinks, snacks, and activities available to them as they wait for the optimization process to conclude.

Across the three subjects with noisy channels, there were a total of four noisy channels, all of which were co-located positions. Although this could be attributed to the inability to maneuver hair in the medial PFC region for certain subjects, due to the probe’s space constraints, a solution is needed for future HD-fNIRS-EEG studies that could encounter the same challenges. One resolution to the space constraints is the additional step of maneuvering hair between the optodes and electrodes before any optimization, as mentioned in the signal quality Sec. 5.2. If using a 3D-printed NinjaCap, another solution would be designing grommets that still maintain support for the optodes and electrodes but are small enough to leave space for hair maneuvering. The space constraints also highlighted the need to find alternative methods for investigating EEG signal quality, as reducing impedance to <10  kΩ did not prevent additional causes of poor EEG signal quality.

### 4.4 fNIRS Stroop Results

Previous research shows that the main neural generators for the Stroop task are the anterior cingulate cortex (ACC) and prefrontal cortex (PFC),^33^^,^^43^^,^^44^ with the hemodynamics localized to the dlPFC and ventrolateral prefrontal cortex (vlPFC).^43^^–^^46^ In these regions, oxygenated hemoglobin is expected to have higher amplitude during incongruent than congruent Stroop tasks. Some studies have shown deactivation in the default mode network (DMN), part of which is located in the medial PFC, as attention-demanding tasks, such as the Stroop task, are conducted.^46^^,^^61^^,^^62^ Although the modified Stroop task used in this study has incongruence in both the easy (choosing between a congruent and incongruent option) and hard (having multiple levels of incongruence in the options) levels, the easy level is comparable to a congruent condition and the hard level is comparable to an incongruent condition, as the general idea for cognitive load tasks is to increase mental effort and attention. In the channel-space results, the easy level shows a deactivation of oxygenated hemoglobin in the region of interest—which is not typically expected in a Stroop task—a potential explanation could be the relative simplicity of the easy level. Participants reported after completing the task that the easy level was extremely straightforward and did not require significant cognitive effort or concentration. Therefore, it is possible that the easy level did not impose sufficient cognitive load to elicit the expected hemodynamic response. In addition, we acknowledge that the hemodynamic response did not consistently return to baseline across all channels. This may be attributed to factors such as physiological noise, motion artifacts, or the timing of the task and inter-trial intervals, which could have influenced the observed signal. Overall, our channel-space results demonstrate a clear difference in hemodynamic activity between the easy and hard levels of the modified Stroop task, with oxygenated hemoglobin increasing from the easy to hard levels in the left and right regions of interest. The lack of statistical significance in HbO responses in channel space may be attributed to several factors: (1) the small sample size (n=13), which limits the statistical power, (2) variability in individual channel responses within the same region of interest, and (3) the task design may have caused a ceiling effect in the hard level condition, as some participants found it easy and performed near maximum accuracy, reducing variability and limiting detectability of condition differences. Despite the lack of significance, the effect size is moderate, suggesting a meaningful trend. Given that the Stroop task and its associated neural activity patterns are well-established, we believe that the qualitative trend observed in our data is still informative. The results from the image reconstruction in AtlasViewer supported previous research by showing a localization of oxygenated hemoglobin in the dlPFC regions, as well as temporal region activation. Temporal region activation has been reported in previous tasks containing linguistic components^43^^,^^63^^–^^66^ and therefore is not unexpected. Deactivation in the medial PFC can also be seen, supporting research surrounding the deactivation of the DMN network when engaging in cognitive tasks. Overall, the channel and image space results support previous research; therefore, these results further validate the co-location method and its use for multimodal imaging.

### 4.5 EEG Stroop Results

Similar to fNIRS, the main neural generators for EEG, regarding the Stroop task, are the anterior cingulate cortex (ACC) and prefrontal cortex (PFC)^67^^,^^68^; however, for EEG studies, the cortical regions of interest differ. In EEG studies, although variable, there are several markers of focus for cognitive load and linguistic tasks, such as the Stroop task. For the more commonly investigated stimulus-locked event-related potentials (ERPs), it is known that the regions of interest are the fronto-central region and the parietal region, with the highest effects expected across the midline (FCz and Pz).^54^ The most noted ERP for cognitive load tasks, such as the Stroop task, is a positive peak around 300 ms to indicate response inhibition,^54^^–^^58^ with P3a being associated with the fronto-central region and P3b being associated with the parietal region.^69^^,^^70^ Some research has shown that with an increase in incongruence, there is a decrease in the amplitude of the P300.^71^ This study focuses on the presence of P3 in the fronto-central and parietal regions. It is expected that the results of this study should be similar to those of previous studies, unaffected by the co-location of optodes and electrodes.

**Fig. 8:**
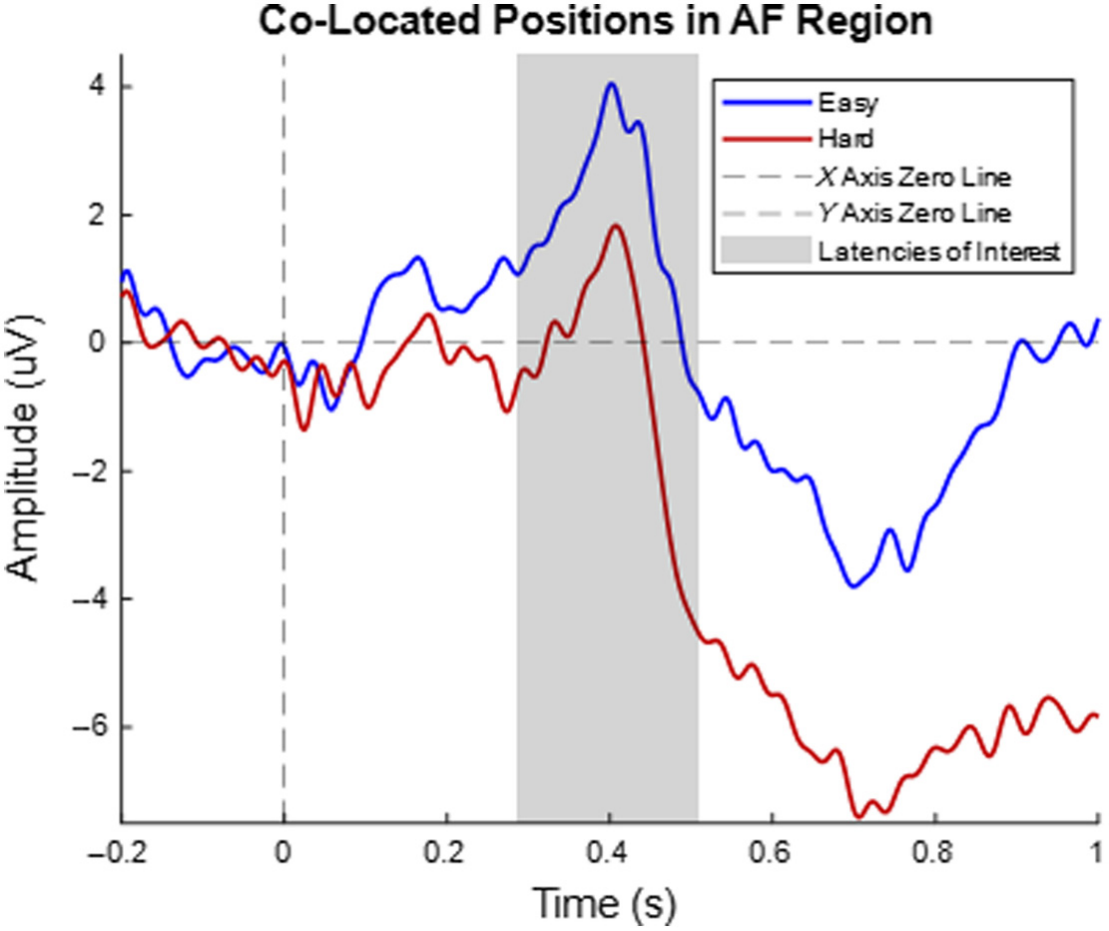
Average ERP of the anterior–frontal region. The anterior-frontal region consists of five electrodes (AF3, AF4, AFz, AFF1h, and AFF2h), all of which are co-located positions. The signal colored in blue represents the easy level, and the signal colored in red represents the hard level of the Stroop task. The dashed black lines indicate the X- and Y-axis zero lines. The shaded region indicates the P3a latency of interest. The t-statistic and effect sizes are −1.308 and 0.413, respectively.

**Fig. 9:**
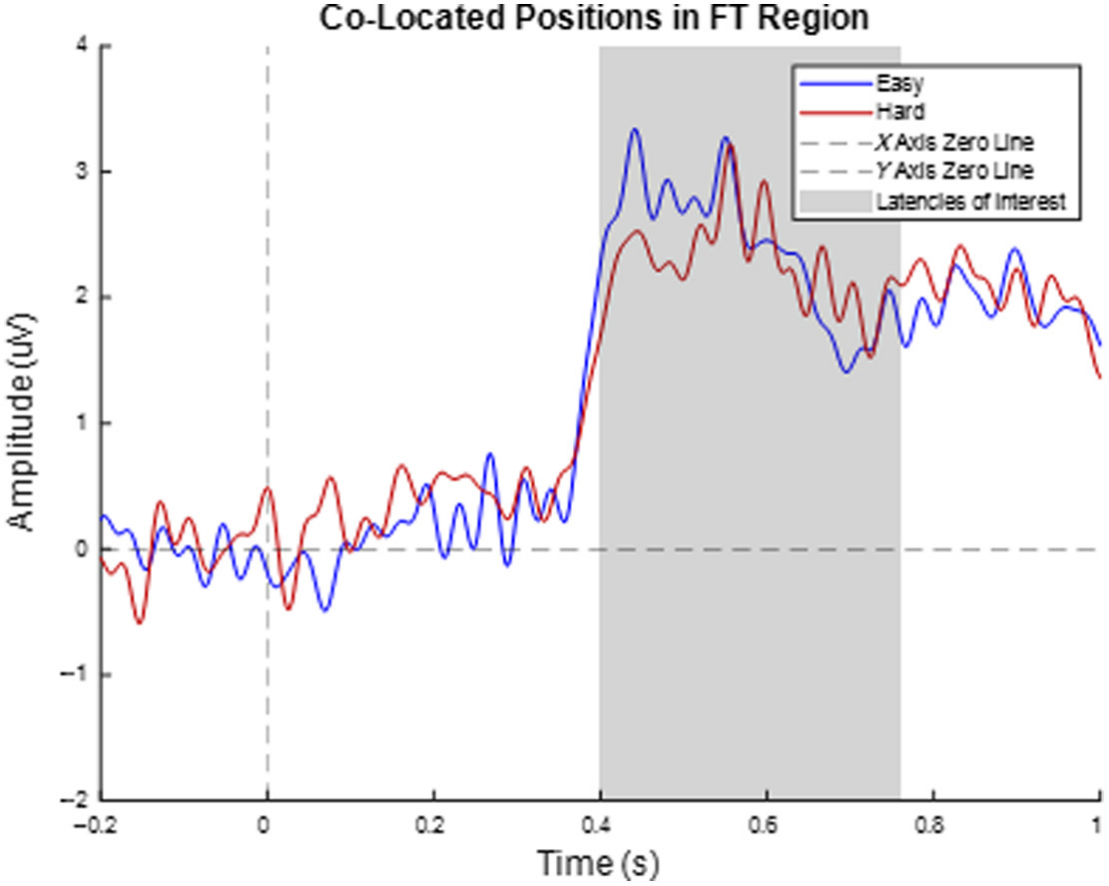
Average ERP of the fronto-temporal region. The fronto-temporal region consists of four electrodes, two per hemisphere (FFT7, FFT8, TTP7h, and TTP8h), all of which are co-located positions. The signal colored in blue represents the easy level and the signal colored in red represents the hard level of the Stroop task. The dashed black lines indicate the X- and Y-axis zero lines. The shaded region indicates the P3a latency of interest. The t-statistic and effect sizes are −0.174 and 0.055, respectively.

Figure 7 shows a multiplot of event-related potentials acquired from the Stroop task at the target electrodes and target regions of interest. For FCz and the fronto-central region, P3a was observed; however, there was little visual difference between the two conditions. For the Pz electrode and the parietal region, P3b was observed, with the incongruent (hard level) condition being more negative than the congruent (easy level). An investigation into the co-located position shows P3a in the average of the five positions in the medial PFC, but at a lower amplitude compared with the target electrode FCz (Fig. 8 in Sec. 6.2). The average of the four electrodes in the fronto-temporal (FT) and temporal-parietal (TP) regions show a P3b response similar to that of the parietal region (Fig. 9 in Sec. 6.2). These results support the presence of P3a in the Stroop task fronto-central region and P3b for the parietal region but are inconsistent regarding expected differences between conditions with differing levels of incongruence for the fronto-central region electrode(s). However, the electrodes from the target electrodes and regions of interest are not co-located; hence, this is not due to any interference on the part of the co-location method. Some studies have shown a P3a component similar to this dataset, such as Boenke et al., where the authors used a modified Stroop task in the form of geometrics,^70^ and a review paper by Polich explains that P300 can vary based on a number of factors, including response time, task type, and the individual subjects in the study.^70^ In addition, the peak amplitudes between the easy and hard levels of this dataset show that numerically, the hard level peaks were lower in magnitude compared with the easy level peaks, which follows the trend of previous research.^71^ The results of the co-located positions in the medial PFC show P3a at a lower magnitude compared with the target electrode and the easy condition has a higher amplitude than the hard condition, both of which are consistent with previous research.^54^^,^^70^^,^^71^ There is little research on the contribution of the FT and TP regions to the Stroop task; hence, little could be concluded about its effect. Regarding statistical analysis, the comparisons between the two conditions are not statistically significant. This could be attributed to the small sample size (n=10). However, between condition statistics of Stroop tasks have previously been reported, where there was partial^56^^,^^58^^,^^72^ or no significant difference between the conditions.^56^^,^^69^^,^^73^ There is also literature that focuses more on the trends, as opposed to the statistics.^55^ Given that previous EEG studies have also reported Stroop effects with partial or no statistical significance, our findings remain consistent with the existing literature. Therefore, we believe that observing the expected qualitative trend in the Stroop task, despite the lack of statistical significance, still supports our conclusion that the hardware development does not alter task-related responses.

## 5 Conclusion

This study shows that our alternative method of co-localizing an fNIRS optode with an EEG electrode can be leveraged for multimodal research, such as sleep, attention, connectivity, and whole-head studies. This method achieves the goal of optimized spatial use of available headspace in multimodal (HD-fNIRS-EEG) imaging, without significantly damaging the signal quality of either system or altering the integrity of the data in the imaging methods involved. In addition to this, this method maintains the benefits of many previous advancements, such as system portability, wearability, modularity, and ease of use for experimenters.

## 6 Appendix: Supplementary Materials

### 6.1 EEG Data Analysis Methods

#### 6.1.1 Stroop Task Analysis

The raw EEG data were analyzed using the MATLAB-based Fieldtrip Toolbox.^53^ First, each run of the subject data was loaded, demeaned, detrended, and bandpass filtered between 1 and 45 Hz, to remove any screen noise and potential interference from other devices. Next, the stimulus-locked trials were defined and any trials with incorrect behavioral responses and trials with a standard deviation greater than 20 or a variance greater than 200 were excluded from the results. Afterward, independent component analysis (ICA) was performed on the continuous version of the data; components for which investigation yielded common noise and motion artifacts were rejected. Finally, the Fieldtrip Toolbox’s ft_rejectvisual function was used to remove any additional trials that no longer passed the visual inspection for ocular or myopic contamination or exceeded the standard deviation and variance parameters. Channels that exceeded the desired parameters were marked as noisy channels and later removed and interpolated by surrounding channels, using the ft_prepare_neighbours and ft_channelrepair functions. Subject data files with more than 13 trials excluded after post-processing (>25% of individual trials rejected per condition) were considered noisy and removed from group analysis. It was determined that there were not enough trials per subject to complete a time-frequency analysis; hence, only an analysis of event-related potentials was completed. For all the included data, the raw, trial-defined data were saved and loaded into a separate code that calculated the average subject ERP using the Fieldtrip Toolbox’s ft_timelockanalysis function for each subject. The code then used the ft_timelockgrandaverage function to average the ERPs from each subject into one group result. Those results were then plotted using the following Fieldtrip Toolbox Functions: ft_SingleplotER, to view individual or average electrodes in the ROI, ft_MultiplotER, to view all electrodes, and ft_Topoplot, to view localization. Based on previous research, the component of interest was P300^54^^,^^55^^,^^57^^,^^58^ and the regions of interest were the fronto-central region and parietal region, with FCz and Pz being the target electrodes in those regions.^54^ To calculate the average ERP across the P3 component, the latency of interest for each subject was calculated for the target electrodes (FCz and Pz) and the target regions of interest: fronto-central (Fz, FCz, FC1, FC2, C1, C2, C3, and C4) and parietal (Pz, P3, P4, CP1, and CP2). The latency of interest was determined similarly to that of conventional latency calculations for EEG.^59^^,^^60^ Significant epochs were defined in a two-part condition: (1) a period during which the difference wave deviated from baseline by greater than 2 standard deviations for longer than 50 ms and (2) provided it exceeded 3 standard deviations in that interval. The beginning of the significant epoch serves as the onset time for the latencies of interest. When the two conditions no longer meet, the 50-ms time point after the last defined significant epoch serves as the offset time for the latencies of interest. A paired t-test was calculated for the onset times, peak amplitude values, and average amplitude across the latency period, for each region of interest (FCz-P3a, Pz-P3b, fronto-central-P3a, and parietal-P3b), using the standard alpha value of 0.05, and the effect size was calculated for the average amplitude across the latency period. Four experimenters conducted the entire analysis process individually, and the resulting plots and statistical analyses were compared, to ensure the integrity of the analysis process.

### 6.2 Supplementary Figures

Figure 8 shows the average trend of the five co-located positions in the anterior frontal region. The trend of these electrodes supports previous research as the average of the five positions exhibit P3a, but at a lower magnitude, compared to the target electrode position, FCz.

Figure 9 shows the average of the four co-located positions (two per hemisphere), located in the fronto-temporal region. The trend appears to follow a trend similar to that of P3b; however, there is not enough literature investigating the ERP behavior in this region, for a Stroop task, to make any concrete conclusions.

**Fig. 10:**
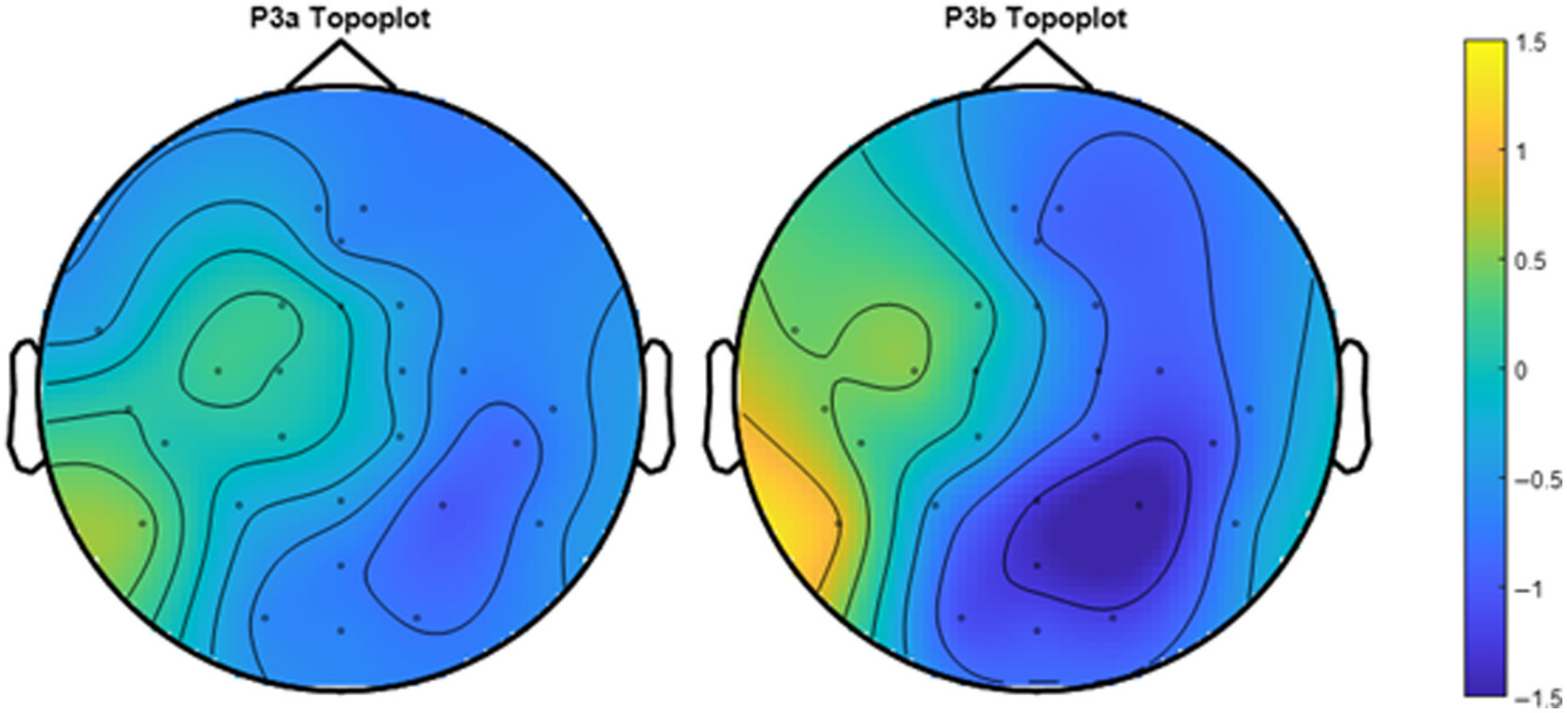
Group topoplot of EEG ERPs. This figure shows the group amplitude of the difference between conditions (hard–easy) across the calculated latency time for P3a (left) and P3b (right), with the noisy channels (AF3, AF4, AFz, and FFT8), mastoids (TP9 and TP10), and EOGs removed. The latency times for P3a and P3b are 286 to 508 ms and 400 to 762 ms, respectively.

Figure 10 shows the group level Topoplot of the EEG ERP difference in conditions (hard-easy), averaged based on their latency period. The P3a (left) plot appears to have a slightly positive amplitude centered on the left side of the fronto-central region and centro-parietal region. The P3b (right) plot appears to have a negative amplitude centered in the parietal region, which is expected because our easy level ERP amplitude has a higher magnitude than that of the hard level, for P3b in the parietal region.

### 6.3 Supplementary Tables

Table 2 shows the log of the power spectral density and corresponding frequencies for the “Induced Crosstalk Present” Graphs in Fig. 3. These results show that there is a consistent peak at 17.4 Hz for the C3 position for the Sources On condition, indicating a presence of crosstalk.

Table 3 shows the log of the power spectral density and corresponding frequencies for the “No Crosstalk Present” Graphs in Fig. 3. These results show that there is a variable peak in our frequency bands of interest (16-18 Hz) for both the Source On and Source Off Conditions, indicating no presence of crosstalk.

Table 4 shows the pruned channels for all fNIRS non-co-located channels. These results show that approximately 2/3 of the channels pruned were not co-located.

Table 5 shows the pruned channels for all fNIRS co-located channels. These results show that overall, approximately 35% of the channels pruned were co-located.

Table 6 shows the results of the t-test between conditions, for the peak ERP amplitude in the EEG results. None of the regions showed a significant difference between conditions.

Table 7 shows the results of a t-test comparing the latency between conditions for the EEG results. None of the regions had a significant difference between conditions.

Table 8 shows the results of the fNIRS channels space t-test from the individual conditions (easy and hard level) against rest. The only results that are statistically significant are the Left and Right DLPFC HbR Easy conditions.

**Table 2:** Crosstalk comparison results: This table shows the log of the power spectral densities and corresponding frequencies, at the individual trial level, for the “Induced Crosstalk Present” graphs in Fig. 3.

Trial	1	2	3	4	5	6
C3 position source on log (PSD) peak power density (${\mathrm{uV}}^{2}/\mathrm{Hz}$)	7.10 * 10e3	7.16 * 10e3	6.52 * 10e3	5.28 * 10e3	6.40 * 10e3	6.88 * 10e3
C3 position source on frequency of peak (Hz)	17.45	17.44	17.43	17.43	17.43	17.43
C3 position source off log(PSD) peak power density (${\mathrm{uV}}^{2}/\mathrm{Hz}$)	1.37 * 10e3	1.08 * 10e3	1.31 * 10e3	1.33 * 10e3	1.67 * 10e3	1.48 * 10e3
C3 Position Source Off Frequency of Peak (Hz)	17.94	16.63	17.93	17.03	16.53	16.66
T7 position source on log(PSD) peak power density (${\mathrm{uV}}^{2}/\mathrm{Hz}$)	1.88 * 10e3	2.63 * 10e3	1.75 * 10e3	1.96 * 10e3	2.14 * 10e3	2.12 * 10e3
T7 position source on frequency of peak (Hz)	16.37	17.64	17.03	17.33	16.53	17.05
T7 position source off log(PSD) peak power density (${\mathrm{uV}}^{2}/\mathrm{Hz}$)	2.98 * 10e3	2.35 * 10e3	1.75 * 10e3	1.95 * 10e3	2.72 * 10e3	1.48 * 10e3
T7 position source off frequency of peak (Hz)	17.05	16.83	17.23	17.82	17.03	17.72
Average non-co-located positions source on log (PSD) peak power density (${\mathrm{uV}}^{2}/\mathrm{Hz}$)	0.84 * 10e3	0.81 * 10e3	0.96 * 10e3	1.01 * 10e3	0.93 * 10e3	0.93 * 10e3
Average non-co-located positions source on frequency of peak (Hz)	16.37	17.84	16.83	17.03	17.13	16.76
Average non-co-located positions source off log (PSD) peak power density (${\mathrm{uV}}^{2}/\mathrm{Hz}$)	1.00 * 10e3	0.92 * 10e3	0.99 * 10e3	1.07 * 10e3	1.03 * 10e3	1.09 * 10e3
Average non-co-located positions source off frequency of peak (Hz)	16.86	17.54	17.93	17.92	17.33	16.28

**Table 3:** Crosstalk comparison results: This table shows the log of the power spectral densities and corresponding frequencies, by subject and location, for the “No Crosstalk Present” graphs in Fig. 3.

Subject	Location	Source on log (PSD) peak power density (${\mathrm{uV}}^{2}/\mathrm{Hz}$)	Source on frequency of peak (Hz)	Source off log (PSD) peak power density (${\mathrm{uV}}^{2}/\mathrm{Hz}$)	Source off frequency of peak (Hz)
SS003	AF8	2.32 * 10e3	17.89	2.05 * 10e3	17.88
SS003	AF4	1.80 * 10e3	16.92	1.98 * 10e3	16.47
SS003	AFz	1.61 * 10e3	16.53	1.29 * 10e3	17.96
SS003	FP1	2.25 * 10e3	17.63	2.61 * 10e3	17.57
SS003	AF3	2.63 * 10e3	16.73	2.52 * 10e3	17.83
SS003	Average of non-co-located positions	0.87 * 10e3	16.27	0.83 * 10e3	17.83
SS004	AF8	1.27 * 10e3	17.88	1.28 * 10e3	16.62
SS004	AF4	0.98 * 10e3	16.75	1.08 * 10e3	17.01
SS004	AFz	0.86 * 10e3	16.75	0.97 * 10e3	17.01
SS004	FP1	0.99 * 10e3	17.88	1.07 * 10e3	17.01
SS004	AF3	0.95 * 10e3	17.35	0.91 * 10e3	17.01
SS004	Average of non-co-located positions	0.38 * 10e3	17.88	0.46 * 10e3	17.01
SS005	AF8	2.30 * 10e3	16.58	2.61 * 10e3	17.09
SS005	AF4	1.96 * 10e3	16.51	2.07 * 10e3	17.09
SS005	AFz	1.97 * 10e3	16.51	1.76 * 10e3	17.09
SS005	FP1	2.37 * 10e3	16.58	2.31 * 10e3	17.09
SS005	AF3	2.26 * 10e3	16.58	1.85 * 10e3	16.58
SS005	Average of non-co-located positions	0.77 * 10e3	16.51	0.81 * 10e3	17.22
SS006	AF8	6.15 * 10e3	17.79	4.94 * 10e3	17.73
SS006	AF4	2.94 * 10e3	17.79	2.90 * 10e3	17.73
SS006	AFz	3.51 * 10e3	16.58	3.25 * 10e3	17.79
SS006	FP1	4.85 * 10e3	17.79	4.48 * 10e3	16.90
SS006	AF3	5.88 * 10e3	16.58	5.99 * 10e3	16.90
SS006	Average of non-co-located positions	1.10 * 10e3	17.34	1.07 * 10e3	16.13
SS007	AF8	2.09 * 10e3	16.40	1.71 * 10e3	16.27
SS007	AF4	1.87 * 10e3	16.86	1.59 * 10e3	16.27
SS007	AFz	1.41 * 10e3	16.40	1.16 * 10e3	16.27
SS007	FP1	1.62 * 10e3	16.40	1.32 * 10e3	16.27
SS007	AF3	1.40 * 10e3	16.86	1.06 * 10e3	16.27
SS007	Average of non-co-located positions	0.57 * 10e3	17.86	0.54 * 10e3	16.93

**Table 4:** Non-co-located channel results: This table shows the number of non-co-located channels that were pruned for each subject, as well as the mean and standard deviation of the group.

Sub #	Total channels pruned	% total channels pruned	Total non-co-located channels pruned	% non-co-located channels pruned
Group average	65 ± 22	27 ± 9	43 ± 16	19 ± 7
SS017	74	31	48	20
SS018	56	24	34	14
SS019	99	42	74	31
SS022	75	32	43	18
SS023	49	21	32	13
SS024	27	11	14	6
SS025	100	42	68	29
SS026	67	28	52	22
SS028	46	19	28	12
SS029	69	29	48	20
SS031	44	18	30	13
SS032	51	21	36	15
SS033	59	25	36	15
SS034	90	38	55	23

**Table 5:** Co-located channel results: This table shows the number of co-located channels that were pruned for each subject, as well as the mean and standard deviation of the group.

Sub #	Co-located channels pruned	% co-located channels pruned	% co-located channels pruned (co-located only)	% pruned co-located channels out of total channels pruned
Group average	22 ± 7	9 ± 3	27 ± 9	35 ± 7
SS017	26	11	31	35
SS018	22	9	27	39
SS019	25	11	30	25
SS022	32	13	39	43
SS023	17	7	20	35
SS024	13	5	16	48
SS025	32	13	37	32
SS026	15	6	18	22
SS028	18	8	22	39
SS029	21	9	25	30
SS031	14	6	17	32
SS032	15	6	18	29
SS033	23	10	22	39
SS034	35	15	42	39

**Table 6:** EEG peak amplitude statistical results: This table shows the group level peaks of the easy and hard level conditions by region and latency, as well as the corresponding paired t-statistic and p-value.

ROI	Latency name	Latency (ms)	Easy-level peak amplitude (uV)	Hard-level peak amplitude (uV)	t-statistic (peak)	p-value (peak)
FCz	P3a	286 to 508	5.56	5.37	0.66	0.52
Pz	P3b	400 to 762	7.11	5.38	1.00	0.33
FC region	P3a	286 to 508	5.19	4.87	0.67	0.51
P region	P3b	400 to 508	5.86	4.58	0.72	0.48
AF region	P3a	286 to 508	4.05	1.55	1.09	0.30
FT region	P3b	400 to 508	3.34	2.84	-0.26	0.79

**Table 7:** EEG onset time statistical results: This table shows the group level onset times of the easy and hard level conditions by region and latency, as well as the corresponding paired t statistic and p-value.

ROI	Latency name	Easy latency onset (ms)	Hard latency onset (ms)	t-statistic (latency)	p-value (latency)
FCz	P3a	294	286	0.41	0.63
Pz	P3b	296	296	1.07	0.31
FC region	P3a	294	288	−0.35	0.73
P region	P3b	296	298	0.71	0.49
AF region	P3a	252	364	−1.32	0.23
FT region	P3b	330	340	0.42	0.68

**Table 8:** Results of the fNIRS channel space t-test. All statistics were calculated using the average hemodynamic response from 10 to 25 s.

ROI	HRF	Condition	t-statistic	p-value	Effect size
Left DLPFC	HbO	Easy	−0.13	0.89	−0.03
Left DLPFC	HbR	Easy	2.48	0.02	0.68
Left DLPFC	HbO	Hard	1.80	0.09	0.50
Left DLPFC	HbR	Hard	0.48	0.63	0.13
Right DLPFC	HbO	Easy	−0.74	0.47	−0.20
Right DLPFC	HbR	Easy	2.38	0.03	0.66
Right DLPFC	HbO	Hard	1.59	0.13	0.44
Right DLPFC	HbR	Hard	−0.68	0.50	−0.19

## Data Availability

The data used in this work is de-identified according to the guidelines of the Institutional Review Board of Boston University and will be provided upon request. All relevant code is available at https://github.com/dejar14/Co-location-Method-fNIRS-EEG- with open public access.
